# Medium term water deficit elicits distinct transcriptome responses in Eucalyptus species of contrasting environmental origin

**DOI:** 10.1186/s12864-017-3664-z

**Published:** 2017-04-07

**Authors:** Antanas V. Spokevicius, Josquin Tibbits, Philippe Rigault, Marc-Alexandre Nolin, Caroline Müller, Andrew Merchant

**Affiliations:** 1 School of Ecosystem and Forest Sciences, University of Melbourne, Creswick, Victoria 3363 Australia; 2 Victorian AgriBiosciences Centre, La Trobe University R&D Park, 1 Park Drive, Bundoora, Victoria 3083 Australia; 3 GYDLE, 101-1332 Av. Chanoine Morel, Québec, QC G1S 4B4 Canada; 4 Faculty of Agriculture and the Environment, The University of Sydney, Sydney, 2006 Australia

**Keywords:** *Eucalyptus*, Transcriptomic, Water deficit, Homeostasis, Ecotype

## Abstract

**Background:**

Climatic and edaphic conditions over geological timescales have generated enormous diversity of adaptive traits and high speciation within the genus *Eucalyptus* (L. Hér.). Eucalypt species occur from high rainfall to semi-arid zones and from the tropics to latitudes as high as 43°S. Despite several morphological and metabolomic characterizations, little is known regarding gene expression differences that underpin differences in tolerance to environmental change. Using species of contrasting taxonomy, morphology and physiology (*E. globulus* and *E. cladocalyx*), this study combines physiological characterizations with ‘second-generation’ sequencing to identify key genes involved in eucalypt responses to medium-term water limitation.

**Results:**

One hundred twenty Million high-quality HiSeq reads were created from 14 tissue samples in plants that had been successfully subjected to a water deficit treatment or a well-watered control. Alignment to the *E. grandis* genome saw 23,623 genes of which 468 exhibited differential expression (FDR < 0.01) in one or both ecotypes in response to the treatment. Further analysis identified 80 genes that demonstrated a significant species-specific response of which 74 were linked to the ‘dry’ species *E. cladocalyx* where 23 of these genes were uncharacterised. The majority (approximately 80%) of these differentially expressed genes, were expressed in stem tissue. Key genes that differentiated species responses were linked to photoprotection/redox balance, phytohormone/signalling, primary photosynthesis/cellular metabolism and secondary metabolism based on plant metabolic pathway network analysis.

**Conclusion:**

These results highlight a more definitive response to water deficit by a ‘dry’ climate eucalypt, particularly in stem tissue, identifying key pathways and associated genes that are responsible for the differences between ‘wet’ and ‘dry’ climate eucalypts. This knowledge provides the opportunity to further investigate and understand the mechanisms and genetic variation linked to this important environmental response that will assist with genomic efforts in managing native populations as well as in tree improvement programs under future climate scenarios.

**Electronic supplementary material:**

The online version of this article (doi:10.1186/s12864-017-3664-z) contains supplementary material, which is available to authorized users.

## Background

Changes in climate over geological timescales have played a major role in the evolution of the Australian vegetation. Northward continental drift, beginning in the late Oligocence (38–23 Ma), led to aridity cycles of increasing severity and duration during the Miocene (25–10 Ma) which have promoted a range of adaptive traits characteristic of the Australian vegetation. Isolation of gene pools via developmental, climatic and edaphic factors, coupled with high degrees of outcrossing has promoted speciation among many Australian plant families, most notably the genus *Eucalyptus* L’Hér genus giving rise to >900 species. The Genus Euclayptus is widely distributed across Australia in tropical, arid, temperate and Mediterranean climatic zones occupying an extensive ecological range from regions with annual rainfall as low as 250 mm [14], to the wet sclerophyll forests of eastern and south-western Australia where rainfall exceeds 1500 mm year^−1^.

Among a range of edaphic and climatic factors governing species distributions, water availability is a significant determinant of eucalypt growth, survival and productivity (see: [1]). A range of adaptive and acclimation traits to enhance growth and survival under the effects of water deficit are prevalent including anatomical changes in leaf shape and area [55], changes in biomass allocation [47], variable stomatal control [27, 56], differences in cell wall reinforcement [20, 22], cell wall water storage [51] and cellular osmolarity [9, 21, 33, 35, 40]. These properties act in concert in many eucalypt species, undoubtedly combining to produce high levels of site-specific variation in performance and survival.

The primary function of physiological and chemical adaptations, either by tolerance or avoidance, ultimately serves to maintain cellular function. The scope of environmental conditions tolerated by the genus *Eucalyptus*, and the diversity of physiological responses to the availability of water detected among contrasting taxonomic groups [5] suggests both isohydric and anisohydric approaches to plant growth and survival are prevalent within the Genus. The concomitant effects of water deficit, such as redox imbalances within photosynthetic reactions or the capacity to dissipate excessive leaf temperatures require the enhancement of homeostatic mechanisms to maintain cellular function.

The complexity and interrelatedness of biochemical and physiological pathways of plants presents a major challenge to our ability to study plant scale responses. More recent technological advances to collect network scale molecular data [13] have led to progress in the adoption of a ‘systems biology’ approach (e.g. [26]) to the study of plants that is still being defined [58]. In addition to the scope of challenges faced by ‘omic’ approaches to comprehensively encompass spatio-temporal variation, interpretation of such datasets is challenging as the development of suitable bioinformatic tools lags behind our ability to acquire data, especially for non-model species. Emerging technologies in bioinformatics to enable a systems approach to multidisciplinary investigations will undoubtedly prove vital in the development of ‘plant systems biology’ and the investigation of dynamic systems from the gene to the landscape scale.

For *Eucalyptus*, understanding physiological and chemical responses of species is presently based on stochastic investigations of pathways that are part of a larger dynamic network. The molecular basis of acclimation offers a logical reductionist approach to investigating network complexity and is now receiving significant attention (e.g. [15, 39]). In plant systems, transcriptional regulation of pathways governing a cascade of chemical and physiological response mechanisms underpinning homeostasis are typically considered in isolation with little context to more general adjustments in metabolism and growth. Investigations collecting ‘omic’ data (for review see: [13]), such as the ‘transcriptome’, therefore offers considerable advantages to guide physiological investigations and provide upstream information on regulatory processes in a broader context of plant scale homeostasis. Whilst it is well known that quantitative and qualitative regulation of plant metabolism exists throughout the framework, transcript libraries offer a logical ‘first step’ in developing higher-level understanding of whole plant responses to environmental change.

Despite a broad understanding of physiological and chemical traits in *Eucalyptus* species conferring acclimation to the effects of aridity, less is known regarding the molecular basis of such traits. More recent investigations at both the genomic (e.g. [15]) and transcriptome level [54] are proving highly informative for our understanding of ‘omic’ responses by eucalypts in adapting to and mitigating against changing growth conditions. Studies into water stress response in model and crop species, which are generally short lived annuals with low tolerance to water deficit, tend to focus on identifying early gene expression changes that trigger the initial responses to a change in water status (e.g. early response transcription factors). In contrast, eucalypt species are long lived perennials thus subjected to seasonal changes in water availability that can vary in severity from year to year depending on climatic zones and prevailing weather conditions [31]. Tolerating such temporal variability and amplitude of osmotic potentials [4, 31, 57] is crucial for survival and reproduction.

Adopting a comparative transcriptomics approach (e.g. [3]), though a combination of experimental design, next generation sequencing and novel bioinformatics approaches, we investigate the gene expression profiles of two *Eucalyptus* species of contrasting taxonomy under controlled and quantified medium term water deficit treatment in order to understand the gene sets that become stably established in responses to prolonged water stress in each species. The species used in this study, *Eucalyptus globulus* Labill. and *Eucalyptus cladocalyx* F. Muell., have contrasting properties for tolerating water deficit with *E. globulus* occupying more ‘mesic’ and *E. cladocalyx* more ‘xeric’ environments [35] with differences in isohydric/anisohydric behaviour observed in traits such as stomatal control [35] and both qualitative and quantitative chemical traits for homeostatic regulation of the cellular environment [32, 34]. Despite these differences, both species are anatomically similar in leaf and plant structure. On this basis, this comparative transcriptome study facilitates an investigation of transcriptome wide differential gene expression on a background of *Eucalyptus* responses to the effects of medium term water deficit to provide insight into the molecular mechanisms that differentiate the ‘mesic’ and ‘xeric’ ecotypes.

## Results

### Leaf level physiological measurements indicate acute water deficit responses

Leaf gas exchange in control (WW) plants was consistent for both species throughout the study compared to severe stress (SS) treated plants, with average net carbon assimilation values higher for *E. globulus* than for *E. cladocalyx* (Table 1)*.* Both net carbon assimilation and stomatal conductance reduced with treatment intensity. Sub-stomatal carbon concentration (c_i_), similarly reduced with treatment indicating that limitations to carbon assimilation are largely attributable to stomatal limitations to diffusion rather than reductions in carboxylation arising to biochemical factors (Table 1). From these results it is clear that the main limitation is water availability in line with the treatments applied.

**Table 1:** Leaf level physiological measurements indicate acute water deficit responses due to treatment

Species	Sample time	Treatment	A (μmol CO_2_ m^−2^ s^−1^)	Post hoc	gs (mol H_2_O m^−2^ s^−1^)	Post hoc	Assimilation weighted c_i_	Post hoc
*E. globulus*	Day 1	WW	15.09 ± 2.21	a	0.512 ± 0.099	a	319.5 ± 20.7	a
		SS	12.96 ± 2.39	a	0.515 ± 0.132	a	330.1 ± 22.8	a
	Day 14	WW	10.98 ± 2.33	a	0.445 ± 0.047	a	322.2 ± 30.4	a
		SS	3.14 ± 2.86	b	0.034 ± 0.037	b	230.2 ± 18.7	b
	Day 28	WW	10.49 ± 3.74	a	0.507 ± 0.071	a	339.8 ± 20.1	a
		SS	1.47 ± 0.82	b	0.012 ± 0.004	b	236.5 ± 32.1	b
*E. cladocalyx*	Day 1	WW	4.76 ± 0.69	a	0.292 ± 0.065	a	354.6 ± 9.2	a
		SS	4.29 ± 0.84	a	0.151 ± 0.042	b	345.1 ± 13.7	a
	Day 14	WW	3.70 ± 0.99	a	0.261 ± 0.057	a	363.0 ± 7.2	a
		SS	1.93 ± 0.31	b	0.034 ± 0.009	b	298.0 ± 16.9	a
	Day 28	WW	4.78 ± 1.10	a	0.176 ± 0.035	a	331.7 ± 11.21	a
		SS	0.62 ± 0.30	b	0.009 ± 0.003	b	249.9 ± 19.1	b

Net CO_2_ assimilation (A), stomatal conductance (g_s_) and assimilation weighted sub stomatal carbon concentration (ci) for *E. globulus* and *E. cladocalyx* seedlings subjected to 8 weeks water deficit (SS) treatment and well watered (WW) control conditions. Post hoc column indicates whether values measured were statistical different (a, b) or not (a, a)

### Sequencing data

**Fig. 1:**
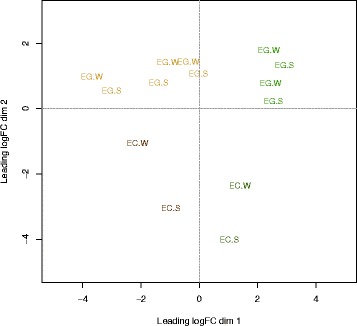
Multi-dimensional scaling plot of transcriptome data distinguishes samples by tissue and species. Multi-dimensional Scaling plot showing the main separation to be between stem derived tissues (*Brown*) and leaf like tissues (*Green*) on the x-axis and species (EG – *Eucalyptus globulus*; EC – *E. cladocalyx*) on the y-axis

Of the 272,167,757 raw reads 240,491,307 passed filtering as high quality (88%; Paired HQ 208,115,303). Of the HQ reads 120,719,749 aligned to 23,623 of the 33,916 annotated *E. grandis* v1.0 genes and these were used in the differential expression analysis. Most of the remaining reads either aligned to the plastid genomes (Stem Tissues Average 327,506 [3.7%]; Leaf Tissues Average 1,876,819 [24.4%]) or to ribosomal genes (Average 2,044,031 [24%]) with on average 85.5% (s.d. 4.1%) of HQ reads explained per sample (Additional file 1: Dataset S1). Investigation of the unmapped reads indicated these largely derived from unannotated ribosomal genes with ~94.7% of reads explained when mapped to the genome sequence as opposed to annotated genes. A measure of the inter-library variation between replicates (reflecting the biological variability in the data) is the common coefficient of biological variation [28] and was estimated as 0.5711 (Gene-wise estimates ranged from 0.321 to 1.803). A multi-dimensional scaling analysis of the transcriptome data (Fig. 1) shows clear discrimination between the two species and between WW and SS treatments with the tissues clustering by type. This high-level view clearly shows that the physiological differences between the species and treatments, as described above, are reflected in the transcriptome and that the isohydric strategy of *E. globulus* is reflected in a lesser transcriptome response in all tissues.

### Transcripts show differential ecotypic responses to water deficit

Differential expression analysis (Additional file 2: Dataset S2) aimed to identify genes showing a general response to treatment, and secondly, to identify genes with ecotype specific responses to treatment. In all cases a stringent FDR of 0.01 was used to determine significance. In total 460 genes showed a significant differential expression response to treatment, with 188 having a higher expression in the SS treatment and 272 having higher expression in the WW control. Of the 460 genes, 141 displayed a significant tissue specific response with 69% in *E. cladocalyx* and 71% in *E. globulus* exhibiting a stem tissue response.

By way of validation we compared the set of differentially expressed genes discovered in this study to those discovered in an independent water deficit experiment where similar stress and well watered treatments were applied to biological replicate plants from the same two species. As only leaf tissues were assayed in the validation study we would only expect to see validation of genes that showed a leaf tissue specific response and a global tissue response, approximately half the genes detected. Also, as the two studies differed in the level of replication we would expect a difference in power to detect differential expression. However, if the genes reported in this study were largely false discoveries it would be extremely unlikely for us to observe a significant overlap between the detected gene sets and further, the direction and magnitude of changes would also be expected to be uncorrelated. Of the 460 genes detected as significantly differentially expressed in stem and/or leaf tissue in this study (FDR <0.01) 232 genes in the validation study (leaf only) showed a significant differential expression and this change is in the same direction and of a similar magnitude in more than 99% of genes (allowing for experimental scaling; Additional file 3: Figure S1). This validation result strongly supports that the signals reported in this study are true signals and not false discoveries.

**Fig. 2:**
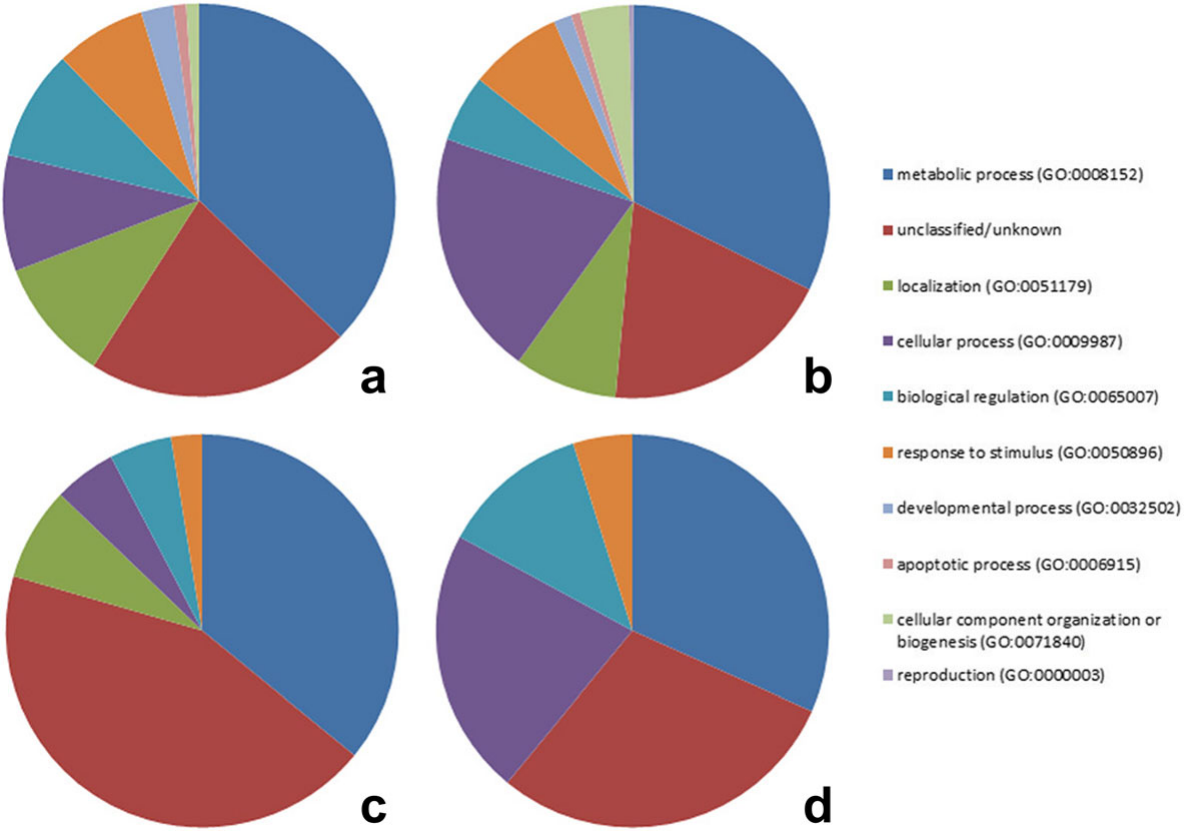
Gene ontology functional classification of differentially expressed genes by treatment and species. Gene ontology functional classifications undertaken using Panther for genes significantly more highly expressed in response to the water deficit (SS) treatment (**a**) and control (WW) (**b**) as well as for genes showing a significant species specific response to water deficit (SS) treatment (**c**) and control (WW) (**d**)

Gene ontology (GO) analysis of biological function using Panther functional classification on genes identified to have an FDR < 0.01 indicated that the majority of the genes more highly expressed in the SS treatment where involved in metabolic processes (37%) or unknown/unclassified (22%), while for the WW control, genes related to metabolic processes (34%) and cellular processes (21%) or where unknown/unclassified (20%) were more highly represented (Fig. 2). AgriGO Singular Enrichment Analysis (SEA) indicated that 24 gene ontologies relating to biological function were significantly enriched (FDR < 0.05) in the SS treatment and included; response to stress, stimulus, abiotic stimulus and water (most highly significant – see Additional file 4: Dataset S3), whereas 38 gene ontologies relating to biological function were significantly enriched (FDR < 0.05) in the WW control and included; flavonoid and phenylpropanoid biosynthetic and metabolic processes (most highly significant – see Additional file 4: Dataset S3).

Highly represented gene families (based on TAIR10 assignment) showing higher expression in the SS treatment included; major facilitator superfamily protein (4), NAD(P)-binding Rossmann-fold superfamily protein (3) and expansin-like B1 (3), whereas gene families highly represented in the WW control included; NB-ARC domain-containing disease resistance proteins (9), disease resistance protein (TIR-NBS-LRR class), putative (6), ankyrin repeat family protein (6), Leucine-rich repeat protein kinase family protein (6), Pathogenesis-related thaumatin superfamily protein (4), basic helix-loop-helix (bHLH) DNA-binding superfamily protein (4), bifunctional inhibitor/lipid-transfer protein/seed storage 2S albumin superfamily protein (4), Subtilase family protein (3), RAD-like 6 (3), P-loop containing nucleoside triphosphate hydrolases superfamily protein (3) and germin (3).

Regarding the ecotype specific response to the treatment, 80 genes showed a significant effect and included; 33 genes that showed significantly higher expression in *E. cladocalyx* in the SS treatment, 41 a significantly higher expression in this species in the WW control, and six, a significantly higher expression in *E. globulus* in the WW control (Table 2, Additional file 2: Dataset S2). No genes showed significantly higher expression in *E. globulus* due to the SS treatment. Of these genes, 20 displayed a significant tissue specific response with 80% of these genes in both *E. cladocalyx* and *E. globulus* had a stem tissue response to treatment. GO analysis of biological function indicated that the majority of the more highly expressed genes (FDR < 0.01) in the SS treatment where either unknown/unclassified (44%) or involved in metabolic processes (44%) while for the WW control, genes related to metabolic processes (32%), cellular processes (22%) and biological regulation (12%) or unknown/unclassified (29%) were more highly represented (Fig. 2). SEA indicated that 3 gene ontologies relating to biological function were significantly enriched (FDR < 0.05) in the SS treatment and included; response to stress, stimulus and abiotic stimulus (see Additional file 4: Dataset S3), whereas 10 gene ontologies relating to biological function were significantly enriched (FDR < 0.05) in the WW control and included; defence response, programmed cell death, death and cell death (most highly significant - see Additional file 4: Dataset S3).

**Table 2:** Transcripts showing a significant differential ecotypic response to water deficit treatment

FDR value	*E. grandis* annotation (Eucgr_ID)	Putative gene function (TAIR definition)	Arabidopsis annotation (TAIR ID)
E. cladocalyx genes showing significantly higher expression in water deficit (SS) treatment			
2E-06	Eucgr.I02543	#N/A	#N/A
2E-06	Eucgr.E03884	NULL	AT3G09950.1
6E-06	Eucgr.J00089	#N/A	#N/A
2E-05	Eucgr.E00360	Expansin-like B1	AT4G17030.1
3E-05	Eucgr.I00610	Regulator of Vps4 activity in the MVB pathway protein	AT4G35730.1
9E-05	Eucgr.I02395	Dehydrin family protein	AT5G66400.1
1E-04	Eucgr.H02812	F-box family protein	AT3G06240.1
3E-04	Eucgr.F00644	NULL	NULL
5E-04	Eucgr.E00358	Expansin-like B1	AT4G17030.1
6E-04	Eucgr.E01430	NAD(P)-binding Rossmann-fold superfamily protein	AT2G45400.1
9E-04	Eucgr.C00678	17.6 kDa class II heat shock protein	AT5G12020.1
1E-03	Eucgr.D01888	Osmotin 34	AT4G11650.1
1E-03	Eucgr.A02790	CAP160 protein	AT5G52300.1
1E-03	Eucgr.H00118	AWPM-19-like family protein	AT1G04560.1
2E-03	Eucgr.F00639	NULL	NULL
3E-03	Eucgr.C03896	Beta-galactosidase 12	AT4G26140.1
3E-03	Eucgr.A02982	NULL	NULL
3E-03	Eucgr.H02021	NULL	NULL
3E-03	Eucgr.C04087	NULL	NULL
4E-03	Eucgr.B00093	HVA22 homologue D	AT4G24960.1
4E-03	Eucgr.B02479	Isovaleryl-CoA-dehydrogenase	AT3G45300.1
5E-03	Eucgr.K02416	NULL	AT1G03910.1
5E-03	Eucgr.F01551	Cytochrome P450, family 721, subfamily A, polypeptide 1	AT1G75130.1
5E-03	Eucgr.B01256	NB-ARC domain-containing disease resistance protein	AT3G14470.1
5E-03	Eucgr.D01823	1 beta-hydroxylase 1	AT4G25700.1
6E-03	Eucgr.A02983	NULL	NULL
6E-03	Eucgr.K02932	Heavy metal transport/detoxification superfamily protein	AT5G03380.1
6E-03	Eucgr.E03924	Tonoplast dicarboxylate transporter	AT5G47560.1
8E-03	Eucgr.F00839	D-aminoacid aminotransferase-like PLP-dependent enzymes superfamily protein	AT4G27190.1
9E-03	Eucgr.B02321	Cytochrome P450, family 707, subfamily A, polypeptide 2	AT2G29090.1
9E-03	Eucgr.B01170	NULL	NULL
9E-03	Eucgr.C02083	Inositol requiring 1-1	AT5G24360.1
9E-03	Eucgr.H03401	NULL	NULL
E. cladocalyx genes showing significantly higher expression in control (WW)			
4E-08	Eucgr.C03357	Proline extensin-like receptor kinase 1	AT3G24550.1
3E-07	Eucgr.K00060	Histidine kinase 3	AT1G27320.1
4E-07	Eucgr.G01856	Disease resistance protein (CC-NBS-LRR class) family	AT1G12290.1
7E-07	Eucgr.B01068	NULL	NULL
5E-06	Eucgr.G00716	NULL	NULL
8E-06	Eucgr.G00693	NB-ARC domain-containing disease resistance protein	AT4G26090.1
2E-05	Eucgr.F03323	NB-ARC domain-containing disease resistance protein	AT4G27220.1
2E-05	Eucgr.E02365	Argonaute family protein	AT2G27040.1
6E-05	Eucgr.H01095	Glycosyl hydrolase 9B13	AT4G02290.1
7E-05	Eucgr.C02074	Disease resistance protein (TIR-NBS-LRR class), putative	AT1G27180.1
8E-05	Eucgr.B03139	NULL	AT1G69160.1
1E-04	Eucgr.J00985	TRICHOME BIREFRINGENCE-LIKE 36	AT3G54260.1
2E-04	Eucgr.J02446	Pathogenesis-related thaumatin superfamily protein	AT4G38660.1
4E-04	Eucgr.C03134	Homeodomain GLABROUS 2	AT1G05230.1
4E-04	Eucgr.G00728	NB-ARC domain-containing disease resistance protein	AT4G27190.1
4E-04	Eucgr.B00934	NULL	NULL
4E-04	Eucgr.G02885	Soybean gene regulated by cold-2	AT1G09070.1
5E-04	Eucgr.K00184	Gibberellin 20 oxidase 2	AT5G51810.1
5E-04	Eucgr.G02880	Soybean gene regulated by cold-2	AT1G09070.1
6E-04	Eucgr.D02419	NULL	NULL
1E-03	Eucgr.E02922	Disease resistance protein (TIR-NBS-LRR class), putative	NULL
1E-03	Eucgr.B00168	Pectin lyase-like superfamily protein	AT5G17680.1
1E-03	Eucgr.E02195	NULL	AT4G24780.1
1E-03	Eucgr.G00729	NULL	NULL
3E-03	Eucgr.K02098	Stomagen	AT4G12970.1
3E-03	Eucgr.A00170	Ankyrin repeat family protein	AT1G03670.1
4E-03	Eucgr.I02127	#N/A	#N/A
5E-03	Eucgr.E01171	HXXXD-type acyl-transferase family protein	AT1G31490.1
5E-03	Eucgr.B03742	Cytochrome P450 superfamily protein	AT5G07990.1
5E-03	Eucgr.G02938	Expansin A4	AT2G39700.1
6E-03	Eucgr.G03078	LOB domain-containing protein 21	AT3G11090.1
6E-03	Eucgr.B00859	B-S glucosidase 44	AT3G18080.1
6E-03	Eucgr.I01620	Senescence-related gene 3	AT3G02040.1
7E-03	Eucgr.C03260	Subtilase family protein	NULL
7E-03	Eucgr.G01048	NULL	AT2G05920.1
8E-03	Eucgr.E00994	BTB/POZ domain-containing protein	AT5G57130.1
8E-03	Eucgr.C00101	Clp amino terminal domain-containing protein	AT4G08455.1
9E-03	Eucgr.A01603	NULL	NULL
9E-03	Eucgr.E02293	Disease resistance protein (TIR-NBS-LRR class), putative	AT5G17680.1
9E-03	Eucgr.G01683	Cryptochrome-interacting basic-helix-loop-helix 1	AT4G34530.1
9E-03	Eucgr.K02082	Leucine-rich repeat protein kinase family protein	AT2G36570.1
E. globulus genes showing significantly higher expression in control (WW)			
2E-10	Eucgr.H02300	20S proteasome beta subunit G1	AT1G56450.1
8E-06	Eucgr.H02302	20S proteasome beta subunit G1	AT1G56450.1
2E-03	Eucgr.F01172	Endoribonuclease/protein kinase IRE1-like	AT5G57850.1
3E-03	Eucgr.F00613	NB-ARC domain-containing disease resistance protein	AT2G17520.1
5E-03	Eucgr.A02524	NB-ARC domain-containing disease resistance protein	AT3G14470.1
7E-03	Eucgr.E00650	NULL	AT4G23493.1

Genes showing significantly higher species specific expression (FDR < 0.01) in either the water deficit (SS) treatment or control (WW) in both *E. cladocalyx* and *E. globulus* from most to least significant

Of the 33 genes that showed a significant response to the SS treatment, 12 showed an opposing response by species and 21 showed a similar but enhanced response relative to *E. globulus* with 12 of these genes, including the top 3, being unknown. The expansin-like B1 (2) was the only gene family to be represented more than once. In the case of the 41 genes that showed a significant response in *E. cladocalyx* to the WW control, 23 showed an opposite response and 18 showed a similar but enhanced response*.* Ten genes in this group where unknown while other highly represented gene families included; disease resistance protein (TIR-NBS-LRR class) putative (3), NB-ARC domain-containing disease resistance protein (3) and soybean gene regulated by cold-2 (2). For the genes in *E. globulus* that showed a significant response to the WW control, all six exhibited an opposite response when compared to *E. cladocalyx* and comprised of only one unknown gene. The 20S proteasome beta subunit G1(2) and NB-ARC domain-containing disease resistance protein (2) gene families were represented more than once.

### Network analysis reveals key pathways linked to differential responses to water deficit between ecotypes

**Fig. 3:**
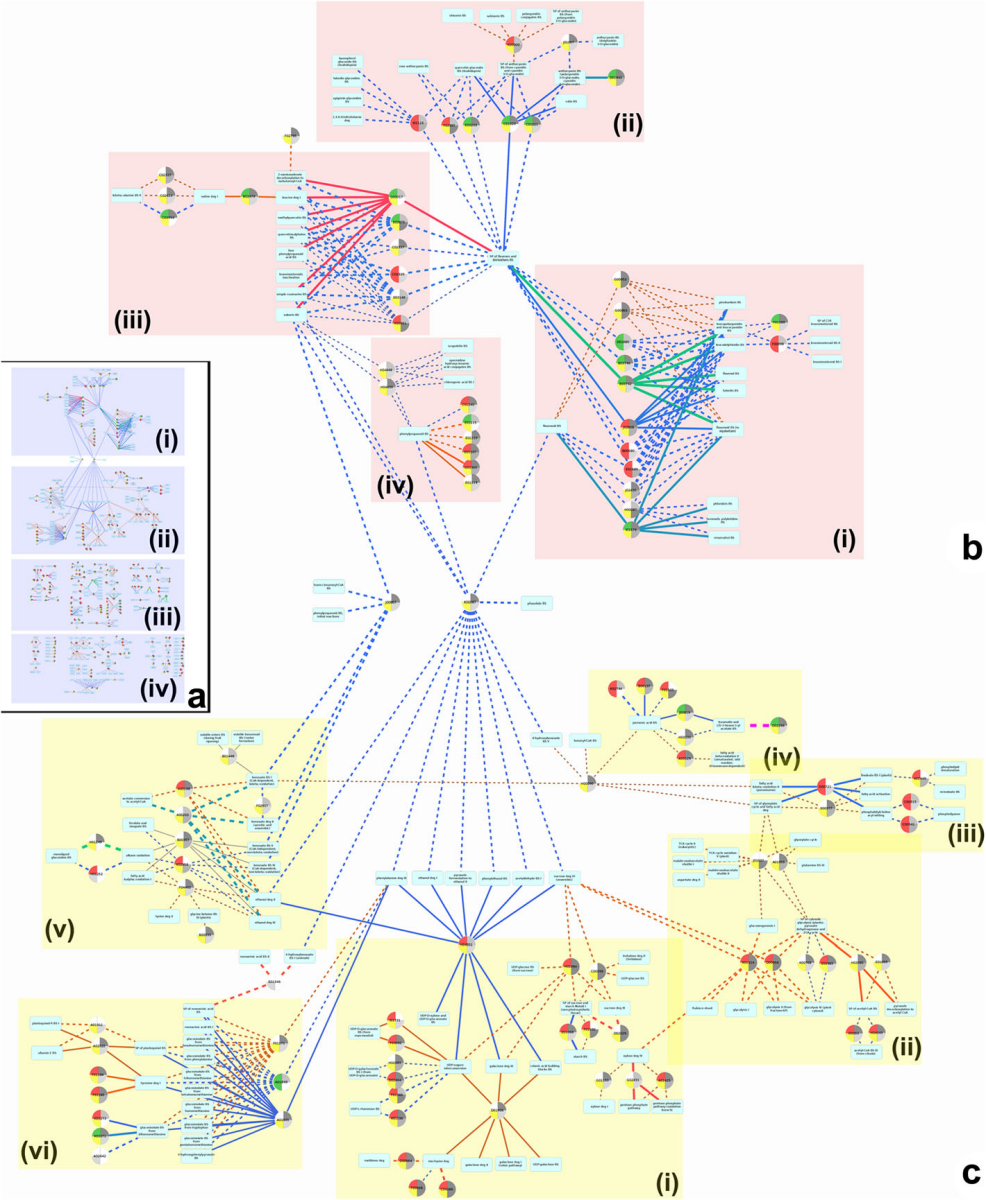
Metabolic pathway network analysis of differentially expressed genes in response to water deficit treatment. Cytoscape graph depicting genes with an FDR of <0.2 with known links to pathways in plant metabolic networks. Genes and pathway interactions are found in two main clusters, cluster **﻿a** (a, i), and cluster **b** (a, ii) as well as numerous small ‘unlinked’ clusters (a, iii and iv). Sub-clusters are also identified within cluster **a** (**b** **i, ii, iii, iv**) and cluster **b** (**c** i, ii, iii, iv, v, vi)

A network analysis of treatment responsive genes (FDR < 0.2) and of known pathways using Cystoscape™ revealed two main linked clusters; Cluster A, comprising 42 genes linked to 42 pathways, and cluster B, comprising of 63 genes linked to 89 pathways, as well as approximately 70 small unlinked clusters containing between 1 and 12 genes (178 in total) with links to between 1 and 14 pathways (165 in total) (Fig. 3a). Due to the complexity of these networks, a brief description of the key observations for the main clusters is detailed below with the low resolution figures provided (Fig. 3 and Additional file 5: Figure S2) being for demonstrative purposes as it is not possible to present the extent of detail in this format. Those wanting to view additional details and/or undertake further analysis of the network presented here are encouraged to download the cytoscape V3.3.0, (http://www.cytoscape.org/) and view the Cytoscape file (Additional file 6: Dataset S4) and interpretation instructions (Additional file 7).

### Cluster A

Cluster A (Fig. 3ai, b) contained 12 genes from 7 families that showed a response (FDR < 0.2) to the SS treatment e.g. more highly expressed, compared to 26 genes from 14 gene families that showed a response in the WW control. Eighteen of the 42 pathways showed differential gene responses to treatment, e.g. at least one linked gene showing higher expression in the SS treatment and WW control, of which 12 genes showed a higher response to the SS treatment and 23 genes in response to the WW control. Four pathways showed an exclusive response to the SS treatment only and were linked to one gene, whereas 23 pathways showed an exclusive response to the WW control and were linked to 19 genes.

Pathways represented in cluster A included sub-clusters relating to; aromatic polyketides, brassinosteroid, flavonoid, flavonol, flavone, leucodelphinidin, leucopelargonidin, leucocyanidin, luteolin, phloridzin, pinobanksin and resveratrol biosynthesis (Fig. 3bi), luteolin, kaempferol, quercitin, apigenin glycoside as well as anthocyanin, pelargonidin, rutin, salvianin and shisonin biosynthesis (Fig. 3bii), beta alanine, phenylpropanoid acid, methylquercetin, quercetinsulphate, coumarins, and suberin biosynthethesis as well as leucine and valine degradation (Fig. 3biii) and phenylpropanoid, spermidine hydroxycinnamic acid, chlorogenic acid and scopoletin biosynthesis (Fig. 3biv). Genes represented more than once in these clusters in are listed in Table 3.

**Table 3:** Highly represented gene families identified in metabolic pathway network analysis

Cluster	Sub-cluster	Putative gene function (Tair10 definition)	Number of gene family members present in sub-cluster	Number of these gene family members more highly expressed in SS treatment	Number of these gene family members more highly expressed in the WW control
A	i	p450 cytochrome	6	-	6
A	i	2-oxoglutarate and Fe(II)- dependent oxygenase	2	2	-
A	i	Chalcone and stilbene synthase	2	-	2
A	ii	UDP-glucosyl transferase	6	-	6
A	iii	O-methyltransferase	4	1	3
A	iii	P-loop containing nucleoside triphosphate	2	-	2
A	iii	ATP-dependent caseinolytic (Clp) protease/crotonase	2	2	-
A	iv	Cinnamyl-alcohol dehydrogenase	4	4	-
A	iv	Caffeoyl-CoA 3-O-methyltransferase	2	-	2
B	i	Rhamnose biosynthesis	2	-	2
B	i	UDP-D-glucuronate 4-epimerase	2	-	2
B	ii	Phosphofructokinase	3	1	2
B	ii	ATP-citrate lyase	2	-	2
B	iii	AMP-dependent synthetase and ligases	2	1	1
B	iii	Phospholipases	2	-	2
B	iv	AMP-dependent synthetase and ligase	2	-	2
B	v	Aldehyde dehydrogenase	4	2	2
B	v	Acyl-activating enzyme	2	1	1
B	v	UDP-glucosyl transferase	2	-	2
B	vi	Tyrosine transaminase	3	2	1
B	vi	2-oxoglutarate (2OG) and Fe(II)-dependent oxygenase	3	-	3
B	vi	Homogentisate 1,2-dioxygenase	2	2	-

Gene families more highly represented in sub-clusters within cluster A and B identified as part of the network pathway analysis in Cytoscape (Fig. 3)

Within this cluster, nine genes showed a different response to treatment at a species level, (solid lines indicating species treatment interaction FDR <0.2) (Fig. 3b). Four of these genes showed a differential response, e.g. a greater relative change in expression between treatments in one species, in the SS treatment and five in the WW control where in all cases the change was observed in *E. cladocalyx*. Two genes showed a significant differential response to the treatment (FDR < 0.05) in the leaf tissue of *E. cladocalyx* (one in the SS treatment and one in the WW control) which were linked to nine different pathways (Table 4).

**Table 4:** Gene and associated metabolic pathways showing significant response to water deficit treatment in *E. cladocalyx*

FDR	*E. grandis* annotation (Egra_ID)	Putative gene function (Tair10 definition)	TR	TiR	Gene and pathway links
Cluster A					
0.004	Eucgr.B02479	Isovaleryl-CoA-dehydrogenase	SS	Leaf	Leucine, valine degradation.
0.005	Eucgr.B03742	Cytochrome P450	WW	Leaf	Flavonoid, flavonol, flavone, leucodelphinidin, leucopelargonidin, leucocyanidin, luteolin biosynthesis.
Cluster B					
0.014	Eucgr.F01185	Homogentisate 1,2-dioxygenase genes	SS	Stem	Tyrosine degradation.
0.047	Eucgr.F01184	Homogentisate 1,2-dioxygenase genes	SS	Stem	Tyrosine degradation.
0.015	Eucgr.I01731	Glucuronokinase	SS	Stem	UDP-glucuronate biosynthesis, UDP-sugars interconversion.
0.025	Eucgr.D01906	UDP-D-glucose/UDP-D-galactose 4-epimerase	SS	Both	Colonic acid, UDP-galactose biosynthesis and UDP-sugars interconversion and galactose, stachyose degradation.
0.047	Eucgr.F03092	Myo-inositol oxygenase	SS	Stem	UDP-glucuronate biosynthesis and UDP-sugars interconversion.
0.02	Eucgr.J0819	Lipoxygenase	WW	Leaf	Traumatin, hexenyl acetate, jasmonic acid biosynthesis.
0.029	Eucgr.K03271	2-oxoglutarate (2OG) and Fe(II)-dependent oxygenase	WW	Leaf	Glucosinolate biosynthesis.
0.040	Eucgr.K03273	2-oxoglutarate (2OG) and Fe(II)-dependent oxygenase	WW	Stem	Glucosinolate biosynthesis.
0.041	Eucgr.E01068	UDP-glycosyltransferase gene	WW	Stem	Starch biosynthesis, sucrose, starch metabolism.
Minor clusters					
0.003	Eucgr.C03896	Beta-galactosidase	SS	Both	Lactose degradation.
0.006	Eucgr.D01823	Beta-hydroxylase	SS	Stem	Carotenoid, lutein, zeaxanthin biosynthesis.
0.008	Eucgr.F01172	D-aminoacid aminotransferase-like PLP-dependent enzymes superfamily	SS	Stem	Aminobenzoate, tetrahydrofolate biosynthesis.
0.009	Eucgr.B02321	Cytochrome P450	SS	Leaf	Phaseic acid biosynthesis.
0.015	Eucgr.H03849	Alpha/Beta-hydrolases superfamily	SS	Stem	Triacylglycerol degradation.
0.029	Eucgr.C00201	Trehalose phosphatase/synthase	SS	Stem	Trehalose biosynthesis.
0.0005	Eucgr.K00184	Gibberellin 20 oxidase	WW	Stem	Gibberellin biosynthesis.
0.006	Eucgr.B00859	B-S glucosidase gene	WW	Both	Coumarin biosynthesis.
0.01	Eucgr.B03312	Petin lyase-like superfamily	WW	Both	Homogalacturonan degradation.
0.013	Eucgr.H04642	Cytochrome P450	WW	Stem	Hydroxylation of laurate.
0.014	Eucgr.I01043	Serine carboxypeptidase-like	WW	Both	Sinapate ester biosynthesis.
0.045	Eucgr.E04056	Peroxidase	WW	Both	Bentanidin degradation.

Genes showing a significantly higher species specific expression (FDR < 0.05) linked to metabolic pathway networks in *E. cladocalyx* seedlings subject to either a water deficit (SS) treatment or control (WW) over an 8 week period. Table notes in which treatment (TR) and tissue (TiR) the significant response was observed in

### Cluster B

Cluster B (Fig. 3aii and c) contained 31 genes from 29 gene families that showed highest expression in the SS treatment and 32 genes from 20 gene families that showed a highest expression in the WW control. Of the 89 pathways present, 38 showed differential responses to the treatment, e.g. at least one linked gene showing higher expression in the WW and SS treatment, where 26 genes showed the response in the SS treatment and 32 genes in the WW control. 32 pathways showed a response only in the SS treatment and were linked to 23 genes, whereas 13 pathways linked to 13 genes showed a response in the WW control only.

Pathways represented in cluster B included sub-clusters relating to; colonic acid, UDP-galactose, UDP-galactoronate, GDP & UDP-glucose, UDP-glucuronate, starch, UDP-rhamnose and UDP-xylose biosynthesis, galactose, melibiose, stachyose, sucrose, trehalose and xylose degradation as well as sucrose and starch metabolism, UDP-sugars inter-conversion and pentose phosphate pathways (Fig. 3ci), malate-oxaloacetate shuttle, pyruvate decarboxylation, glycolysis, gluconeogenesis, glyoxylate cycle, Rubisco shunt, TCA cycle, as well as glutamine and acetyl-CoA biosynthesis and aspartate degradation pathways (Fig. 3cii), linoleate and ricinoleate biosynthesis as well as phospholipases, phosphatidylcholine acyl editing, phospholipid desaturation and fatty acid activation, degradation and oxidation pathways (Fig. 3ciii), jasmonic acid, traumatin and (Z)-3-hexen-1-yl acetate as well as fatty acid beta-oxidation pathways (Fig. 3civ), benzoate, ferulate, glycine, monolignal glucoside and sinapate biosynthesis as well as benzoate, ethanol and lysine degradation, acetate conversion to acetylCo-A and alkane and fatty acid oxidation (Fig. 3cv), and 4-hydroxyphenylpyruvate, glucosinolate, plastoquinol, rosmarinic acid and vitamin E biosynthesis as well as tyrosine degradation pathways (Fig. 3cvi). Genes represented more than once in these clusters in are listed in Table 3.

Within this cluster, 18 genes showed a differential response to treatment at a species level (solid lines indicating species treatment interaction FDR < 0.2) (Fig. 3c). Eight genes showed a differential response, e.g. greater relative change in expression between treatments in one species, to the SS treatment and ten to the WW control where, in all but three cases, this response was observed in *E. cladocalyx.* Nine genes showed a significant differential response to the treatment (FDR < 0.05) in mostly the stem tissue of *E. cladocalyx* (five in the SS treatment and four in the WW control) which were linked to 13 different pathways (Table 4).

### Minor clusters

Of the 70 small clusters identified (Additional file 5: Figure S2) 20 showed a significant response e.g. higher expression, as a result of the SS treatment only, 30 as a result of the WW control only and 20 showed responses in both SS treatment and WW control. These clusters comprised 178 genes from 118 gene families where 78, representing a total of 55 gene families, showed a significant response to the SS treatment and 100, representing 63 gene families, showed a significant response to the WW control.

Gene families responsive to the SS treatment that were more highly represented (>3 genes) included; alpha/beta-hydrolases (5), basic chitinases (5), Zinc-binding dehydrogenases (4), AICARFT/IMPCHase bienzymes (3), indole-3-acetate beta-D-glucosyltransferases (3). On the other hand, gene families responsive to the WW control included; cytochrome P450 (4 gene family members represented), glycosyl hydrolases (4), pectin lyases (4), alpha carbonic anhydrase (3), alpha/beta-hydrolases (3), beta-galactosidases (3), carboxyesterases (3), cellulose synthase (3), peroxidases (3) and UDP-glucosyltransferases (3).

Within these 70 clusters, 43 genes showed a notable differential response to treatment at a species level (solid lines indicating species treatment interaction FDR < 0.2) (Additional file 5: Figure S2). Nineteen of these genes showed a differential response, e.g. greater relative change in expression between treatments in one species, in response to the SS treatment and 24 in response to the WW control where in all but five cases, this response was observed in *E. cladocalyx*. Thirteen genes showed a significant differential response to the treatment (FDR < 0.05) in often both the leaf and stem tissue of *E. cladocalyx* (seven in the SS treatment and six in the WW treatment) which were linked to 16 different pathways (Table 4).

## Discussion

This comparative expression profile for *Eucalyptus* species of contrasting ecotype under the effects of medium term water deficit provides insight into the molecular mechanisms that underpin adaptive and acclimation responses. Plant responses to treatment induced a stomatal response with concurrent physiological changes indicating that the main limitation for water deficit treated plants compared to the well watered was water availability. The treatments triggered a cascade of gene expression responses associated with the concomitant effects of water deficit with several patterns in gene expression emerging and offering insight into the comparative stress response mechanisms employed by *Eucalyptus* species. Notably, this study highlights the enhanced transcriptional responses observed in key pathways of species adapted to more water limiting environments.

This investigation demonstrates the importance of a plant scale transcriptomic approach encompassing multiple tissue types. Over 78% of genes identified as responding to the stress treatment occurred in the stem, demonstrating the potential for significant regulatory processes being modulated from this tissue. Previous and current approaches investigating plant responses to resource availability are focused on leaves potentially overlooking significant molecular, chemical and physiological regulation of processes governing stress responses. Such results clearly demonstrate the importance of ‘whole plant’ studies adhering to the literal interpretation of the term.

Encompassing multiple tissue types exacerbates a major challenge to ‘omic approaches – the complexity of interpreting such large volumes of data. The bioinformatic approach outlined here, has successfully highlighted patterns in transcript data and produced interpretable representations of network structure. It is important to recall that our intention for this study is not to comprehensively summarise the entire transcriptome, nor the pathway analysis constructed, but to offer insight into the transcriptional response mechanisms to applied water deficit and provide a framework for further investigation into the molecular, chemical and physiological mechanisms underpinning tolerance to water deficit in the genus.

### The known unknowns

Among the most highly induced transcripts observed in this study, many were of unknown function. Whilst repositories of *Eucalyptus* sequence data are improving rapidly in scope (see for example, [15, 39, 54]) much work remains to complete the functional annotation of the genome, especially given the broad ecotypic scope, chemical composition and diversity of physiological and growth responses within the genus. The targeted experimental and bioinformatic approaches employed here prioritises a small set of genes as candidates for further investigation. This use of contrasting ecotypes has enabled us to distinguish common mechanisms and species-specific responses from the ‘background’ of gene expression necessary for general cellular function. This approach offers great potential for more functional, mechanistic approaches to characterising transcriptome wide responses to the onset of water deficit with implications for both upstream (genomic) and downstream (proteomic, metabolomic) responses.

### Photoprotection and redox balance

Amongst the most immediate challenges to photosynthetic tissues during resource imbalances is avoiding the consequences of excessive photon flux. In this study, several patterns emerge at the network scale pertaining to the maintenance of redox balance. For example, in cluster A, a range of photoprotective response mechanisms from the phosphoenolpyruvate pathways are observed to respond including transcripts governing redox balance, most notably those of several cytochrome P450 enzymes which play a central role in the oxidation of organic compounds [41]. The role of P450s in oxidising organic compounds, or as the terminal of electron transport chains (including that of NADPH) represents significant homeostatic control over redox balance. Such control aids in the preservation of photosynthetic infrastructure and the reactions of primary photosynthesis (by providing reductant for the photosynthetic reactions) thus mitigating excess photochemical energy experienced during times of stress or alternatively in times of excessive photoassimilate production. Similarly, antioxidant systems such as the glutathione and ascorbate pathways appear regulated in cluster A and in the unclustered networks supporting previous studies on the role of antioxidant pathways and redox balance in *Eucalyptus* leaves (e.g. [10, 17]). Sequencing and subsequent annotation of these pathways provides solid evidence for their inducible responses in *Eucalyptus* tissues and together with the induction of P450 enzymes provide a useful basis for further characterisation in maintaining redox balance under conditions requiring quenching of excess photochemical energy or as an indicator of photoassimilate supply to cellular metabolism.

### Phytohormones and signalling molecules

Rapid elicitation of signalling molecules represents upstream regulation of plant processes governing plant scale resource allocation and utilization. A range of genes involved in the synthesis of phytohormones is observed to respond in the present network. Genes encoding phytohormones in the gibberellin, auxin and cytokinin groups are observed, most notably among cluster A and the associated gene analogues encoding photoprotective properties. The wide distribution of phytohormonal responses across the network likely reflects the broad chemical and physiological processes that rely on hormonal regulation in higher plants. Of particular note is the up regulation of genes governing the synthesis of quercitin in *E. cladocalyx*. Quercitin is a strong antioxidant compound [50] that is antagonistic to the functions of the auxin class of phytohormone [12]. Inhibiting the role of auxin may lead to broad changes in resource allocation to the scale of plant growth habit. Such influence may lead to changes in plant scale resource allocation and concomitant traits associated with resource limitation. It is tempting to suggest that the expression of quercitin and its antagonistic effects on a major hormonal signalling pathway may serve to explain at least some of the variation in growth habit and water use observed across the genus [35, 42, 49, 57]. Further investigations of this gene expression pattern and spatio-temporal assessments of transcript and compound abundance may serve to unravel this fundamental process associated with ecotypic variation within *Eucalyptus*.

### Primary photosynthesis and cellular metabolism

A range of pathways and gene analogues governing the cycling of primary photosynthates and respiratory substrates were observed across the network. Combined, these indicate a broad reconfiguration of primary photosynthetic reactions in response to the water deficit treatment. For example, UDP-D galactose-4-epimerase-1 governs the inter-conversion of galactose to glucose primarily for input into glycolysis. This gene shows differential regulation in response to the treatment (Fig. 3ci). UDP-D galactose-4-epimerase-1, producing glucose-1-phosphate, may also provide substrate for alternative pathways, most notably to the synthesis of inositols via inositol 3 phosphate synthase and downstream pathways previously linked to stress tolerance – including that within the *Eucalyptus* genus [2, 4, 32]. Cycling of photoassimilates and subsequent removal from the primary photosynthetic reactions of the cell is thought to alleviate sugar-mediated repression of photosynthesis in plants (e.g. [16, 18, 46]) thus avoiding, at least in part, photo-oxidative damage. A major component of this is the galactosylation of sugars for symplastic loading mechanisms characteristic of many tree species [52] including that of *Eucalyptus* [36, 37, 45]. Gene analogues governing the galactosylation of carbohydrates to form di-, tri- and tetra-saccharides such as melibiose, raffinose and stachyose is observed (Fig. 3ci) with potential implications for rates of carbon export from leaves.

The interconnectedness of pathways within primary metabolism is a major contributor to the capacity for homeostatic regulation of the system to balance inputs such as water and light. It is therefore not surprising that response mechanisms such as redox balance, photoprotection and changes in the cycling of primary photosynthates dominate the response to the water deficit treatment and share commonalities with previous investigations in response to alternative stress types (e.g. [17, 54]). An example of this is the expression of phosphogluconolactonase (Fig 3ci) closely linked with the pentose phosphate pathway. The pentose phosphate pathway may replace glycolysis under suboptimal conditions producing NADPH and a range of 4 and 5 carbon sugars. Products of the pentose phosphate pathway may provide substrate for several biochemical processes such as input back into glycolysis or the shikimic acid pathways for subsequent synthesis of aromatic compounds. Within primary metabolism, the necessity for tight regulation of the network means that subtle differences in transcript abundance may have large consequences on the observed phenotype. It is therefore important to remember that transcript abundance – even that of differentially expressed genes among contrasting ecotypes – is not a sole indicator of the *significance* of the response.

### Secondary metabolism

Previous investigations into secondary metabolites in *Eucalyptus* have illustrated a broad diversity and range of concentrations among leaf oils [23, 24], waxes [25], phenolics [7] and tannins [29, 44] with several efforts to characterise genetic associations that underpin this diversity [19, 43]. In this study, both common and differential response in the expression of candidate genes governing the synthesis of secondary metabolites was observed between the two species. For example, tyrosine transaminase (Fig. 3cvi) is differentially expressed in response to treatment. Tyrosine is a phenolic amino acid thus may act as a precursor to the synthesis of other phenolic compounds or alkaloids [53]. Linkages between the synthesis of tyrosine and the synthesis of compounds known to have homeostatic redox functions such as the vitamin E in close proximity to gene analogues for plastiquinol biosynthesis indicate that redox balance is a major factor in plant acclimation strategies to the effects of imbalances between the availability of water and excessive photon flux. The diversity of secondary metabolism in the genus is reflected within the network compiled and serves as a valuable sequence resource to investigate differentially expressed chemical traits within the genus.

This study reports a novel experimental approach that achieves a comparative investigation of transcriptome responses two *Eucalyptus* species of contrasting ecotype to medium term water deficit. The range of transcript responses among the species highlights the mechanisms employed to maintain cellular function under a contrasting environmental conditions. These patterns, in several cases having the potential to influence broader structural and chemical processes governing adaptation to water deficit, are likely to be reflected in gene expression among a broader range of species within the genus and thus are candidates underpinning inter-species variation. This study highlights the strength of a transcriptomic approach to characterise plant responses in the context of metabolic network scale expression profiles and follow up experiments on more species and a range of time points will enable discovery of the genes that regulate species specific responses to water deficit as well as to the gene sets that define those responses. Compilation of such repositories for transcriptome data will undoubtedly serve as a valuable resource for further investigation at a range of scales into the responses employed by *Eucalyptus* to tolerate such a wide scope of environmental conditions.

## Conclusion

This study describes and annotates an enhanced transcriptional response to water deficit in key pathways and associated genes in a ‘dry’ (xeric) climate eucalypt species adapted to water limited environments compared to a species originating from less arid conditions (mesic). The observed transcriptional response was of a greater magnitude and diversity in stem tissue. Of the differentially expressed genes, a significant proportion were of unknown function, indicating both unique and poorly understood mechanisms underlying the success of members in this genus in water limiting environments. Genes with known function were found in pathways linked to photoprotection and redox balance, phytohormone and long-distance signalling, primary photosynthesis and cellular metabolism. This suggests that the ‘dry’ climate eucalypt species has tightly regulated mechanisms across the metabolic network for broad scale modification of their biochemistry to reduce water loss while coping with the resultant increases in heat and light energy associated with the inability regulate this via modified physiology. Identification of these pathways and associated genes provides the opportunity to further investigate and understand the mechanisms and genetic variation linked to this important environmental response and assist in genomic efforts linked to managing native populations or tree improvement programs.

## Methods

### Plant material and experimental design

Seeds of *E. cladocalyx* were obtained from the Australian Tree Seed Centre at the Commonwealth Scientific and Industrial Research Organisation (CSIRO) and were germinated and raised in a naturally illuminated glasshouse at the University of Melbourne, Creswick (l37.43° S, 143.89° E). Seedlings were germinated in seed raising mix (Osmocote™>). Day/night air temperature was maintained at 20/12 °C and relative humidity at 60/70%. Irradiance as measured at the canopy level was approximately 600 *μ*mol m^−2^ s^−1^ (PPFD) for a 13 h photoperiod (6:00–17:00). After 8 weeks, 60 plants were re-potted into 9 L containers containing a mixed peat/sand 50/50 v/v fertilised with 4 g L^−1^ slow release fertiliser Nutricote-100, 13/13/13 N/P/K with oligo elements, and watered six times daily by drip irrigation to field capacity. Seedlings were grown for 20 weeks prior to experimentation. For *E. globulus*, 60 ramets of clone X46 were sourced Narromine Transplants (Narromine, Australia) and potted into 9 L containers. Seedlings were grown following the same protocols and conditions as *E. cladocalyx* and experimentation took place after 14 weeks of growth in the glasshouse.

For experimentation, 24 *E. cladocalyx* and 36 *E. globulus* seedlings of similar height and leaf area were selected and segregated into two treatments: well watered (WW) and severe water deficit (SS). Treatments were determined gravimetrically based on calculated water use of control plants [30]. SS plants were given 45% of water used by WW to exhaust the reservoir of water in the pot by week 8 of the treatment period.

### Leaf gas exchange measurements

Patterns in leaf gas exchange across the light period were determined on six plants of each species in each treatment using a LICOR 6400 Infra-red gas analyser (LICOR, Lincoln, Nebraska, USA) with a blue-red diode illuminator for leaf-level photosynthesis from 9 am (two hours after the photoperiod began) to 5 pm. A single leaf was selected on each plant and measured sequentially with at least 10 independent measurements taken on each leaf on each day. Fully expanded leaves were chosen for measurement with a different leaf used in each diurnal cycle. Light conditions within the LICOR chamber were set to tracking mode to approximate the growth chamber conditions. CO_2_ mole fraction in the reference air stream was set to 400 μmol mol^−1^ and temperature and relative humidity in the measuring chamber was maintained within the LICOR chamber to approximate ambient conditions. Net CO_2_ assimilation rate (A, μmol m^−2^ s^−1^) as well as stomatal conductance to water vapour (g_s_, mmol m^−2^ s^−1^) were recorded and used to compute the ratio of intercellular to atmospheric CO_2_ concentration c_i_/c_a_.

### Tissue collection

Plant tissues were harvested between 12 and 2 pm on a sunny day at the end of week 8 of the treatment period and placed immediately into liquid nitrogen before being stored at −80 °C prior to RNA extraction. *E. globulus* (EG) tissue sourced was pooled from four separate individuals from the WW and SS trails including Apical Tip (AT), comprising of tissue above the first discernable node of the apical stem, Fully Expanded Leaf (FL), large leaves located in the top 1/3 of the plant that had full exposure to light, Primary Stem (ST), node 2, 3 and 4 of the apical stem and exhibiting a juvenile state (squared shape), Secondary Xylem (XY), xylem tissue scrapped with significant pressure from the surface of the stem using single edge razor blade after phloem removal in stems >2 cm in diameter, and Secondary phloem (PH), the secondary phloem tissue removed from stems >2 cm in diameter when sampling the XY tissue. In total there were 10 different tissue/treatment tissue samples for this species (Additional file 8: Figure S3). *E. cladocalyx* (EC) tissue was sourced from one individual in the WW and SS trails as individuals were not clonally related and included the following tissues; AT, ST and FL, as per *E. globulus* description, except in the case of ST where the first node of a number of lateral shoots was sourced (*E. cladocalyx* did not show strong apical dominance and was highly branched), as well as Secondary Stem (S2), where all xylem (developing and developed) and phloem tissue from a stem >1 cm in diameter was sourced due to difficulties of separating phloem from the xylem in the SS trial. In total, eight different tissue/treatment tissue samples were present for this species (Additional file 8: Figure S3).

### RNA isolation, library construction and sequencing

All tissue samples were ground using an IKA M20 Universal Mill (IKA-Werke, Staufen, Germany) and MM301 Ball Mill (Retsch, Haan, Germany) for 3 min at 25Hz under liquid nitrogen. 3 g of milled tissue was then extracted following protocols outlined in [8] and further purified with RNAzol RT (Molecular Research Centre, Cincinnati, OH, USA) and Zymo-spin IV-HRC spin columns (Zymo Research, Irvine, CA, USA). Following this, RNA quality and quantity was assessed using the Experion™ Automated Electrophoresis System (BioRad, Hercules, CA, USA) and Nanodrop 2000 (NanoDrop Products, Wilmington, DE, USA) indicating sufficient high quality RNA for library construction in all tissue samples except for the EC/SS/AT and EC/SS/ST samples (Additional file 8: Figure S3).

For mSEQ library preparation total RNA was PolyA enriched (Dynabead mRNA purification kit - Invitrogen, Grand Island, NY, USA), sheared by heating and used as a template for cDNA synthesis (Super Script III Reverse Transcriptase - Invitrogen). Second strand synthesis and end polishing was then undertaken using DNA Polymerase I and *E. coli* RNaseH (New England Biolabs (NEB), Ipswich, MA, USA) with the resulting dscDNA cleaned using SPRI beads (Beckman-Coulter, Brea, CA, USA). dA-Tailed using 5 U Klenow Fragment (NEB E6054AA) was undertaken with the tailed dscDNA ligated to Illumina Y junction adaptor fragments using of T4 DNA ligase reaction buffer (NEB) and where again cleaned using SPRI beads. Selected library fragments were then individually barcoded using primers compatible with Illumina sequencing platforms for tracking PCR using Phusion polymerase (Finnzymes, Vantaa, Finland) on a STRATAGENE Mx3005 (Agilent Technologies, Santa Clara, CA, USA). Amplified barcoded libraries were checked for size distribution on a GENECHIP1000 (Agilent Technologies) and titrated by Nanodrop 2000 and qPCR using the KAPA Library Quantification Kit. For a detailed methodology on library preparation please see (Additional file 9). Libraries where equimolar pooled and sequenced on a GAIIX (Illumina San Diego, CA, USA) using v2 chemistry and a standard 100 + 8 + 100 bp sequencing protocol.

### Post sequencing bioinformatics

Fastq sequence files were post run filtered to leave high-quality (HQ) reads using Nuclear version 2.6.11 software (Gydle Inc. http://www.gydle.com/) to a minimum length of 50 bp and a minimum quality score of phred 20. HQ reads were mapped to the *E. grandis* v1.0 genome annotation v1 [39] using Nuclear (minimum HSP-length 60 bp; maximum mismatches per HSP 4; sensitivity 25 bp; minimum percentage covered 70%; Paired read distance maximum 1000 bp; read masking 4; extend masked - yes) and read counts were tallied for each gene model. Differential expression was analysed using R v3.0.1 (http://www.r-project.org) using the edgeR package v3.2.4 [28, 48] with read counts normalised by the model-based scaling normalisation method, Trimmed Mean of M-values (TMM) [59] and the data fit to a negative binomial generalised log-linear model factoring for species, tissue (leaf = FL, AT and stem = S2, ST, XY, PH, Additional file 8: Figure S3) and drought treatments. The model assumes the data are over dispersed with contributions to variance from sampling and the biological sources of variation. Individual gene-wise dispersions were used in all testing to accommodate the over-dispersed relationship between read count and variance (Additional file 10: Figure S4) (for details on the model fitting in edgeR see [28]). Likelihood ratio tests identified genes with statistically significant effects and resulting P-values were adjusted for multiple testing using Benjamini-Hochberg FDR adjustment approach [6].

Information from the plant metabolic network database Plantcyc (http://www.plantcyc.org/ ) and the pathway/genome database Biocyc (http://websvc.biocyc.org/) as well as the Gydle mapping as part of this study were used to visualise gene pathway networks using Cytoscape (V3.1.1, http://www.cytoscape.org/). To create the cytoscape graph, data was converted to triples data, loaded into a triplestore (OpenLink Virtuoso) and then linked via the TAIR identifiers. Sparql queries where then used to connect identified genes from the Gydle mapping to pathways. For network display, identified genes with FDR value of <0.2 for the DroughtW_FDR (response to water deficit) and SpeciesEG_DroughtW_FDR (species specific response to water deficit), were used (Additional file 2: Dataset S2). This modest FDR was chosen to facilitate the development of a suitably representative gene pathway network to support elucidation of responses. To assist with interpretation, additional data on the identified genes was visualised through attributes including graph line colour, style and width as well as gene pie charts found at each node. Details of these attributes can be found in the Additional file 7. Gene ontology functional classification was undertaken using Panther [38] and Singular Enrichment Analysis was undertaken using AgriGO [11] on genes with and FDR < 0.01.

### Validation of transcripts

Validation of differential gene expression was through an independent (unpublished) study of leaf gene expression responses in the same two eucalypt species, treated under a similar medium term water stress regime. Protocols for tissue collection, RNA extraction, sequencing, bioinformatics and analysis were as described above with the exception that library construction targeted total RNA rather than mRNA as in this current study. The analysis compared both stem and leaf tissue from this study to leaf tissue from the validation study where logFC and FDR values where used to compare expression patterns across common genes found in both studies that showed a response to the drought treatment.

## Additional files

Additional file 1: Dataset S1. Spreadsheet containing filter and mapping summary data. (XLS 28 kb)

Additional file 2: Dataset S2. Spread sheet containing the full analysis of the RNAseq data including all 23,623 identified genes (all analysis), genes significantly differentially expressed due to treatment (diff_res_to_treat) and species specific genes with significant differential expression (spp_diff_res_to_treat). (XLSX 13892 kb)

Additional file 3: Figure S1. Plot of log fold change in gene expression for leaf and stem tissues between water deficit (SS) treatment or control (WW) treatment as detected in this experiment (X-axis) versus log fold change in gene expression in leaf tissues between water deficit (SS) treatment or control (WW) treatment (Y-axis) as detected in a follow up water deficit experiment on seven eucalypt species (*N* = 56). Grey (+) symbols represent all genes while red (o) represent genes validated at an FDR of < 0.01 in both experiments. It is clear that validated genes also show responses in the same direction between experiments. (PNG 25 kb)

Additional file 4: Dataset S3. AgriGO Singular Enrichment Analysis outputs. (XLSX 59 kb)

Additional file 5: Figure S2. Small unlinked clusters depicting genes with an FDR of <0.2 with known links to pathways in plant metabolic networks. Small unlinked clusters have been arranged according to whether they show a species specific water deficit response (solid lines), with or without a water deficit only treatment response (dashed lines), (a), or water deficit only treatment response (dashed lines) (b). Clusters have been arranged further into those with a higher expression in response to the water deficit treatment (SS) (i), a differential response to treatment where some genes in a cluster show higher expression in response to water deficit treatment (SS) and others to the well watered control (WW) (ii), or higher expression in response to the well watered control only (WW) (iii). (PNG 2743 kb)

Additional file 6: Dataset S4. The full cyctoscape file to allow for more detailed examination of genes/pathways and how they have responded to the water deficit treatment (SS) and well watered control (WW). To view file, please download the Cytoscape V3.3.0 program free from the following website: http://www.cytoscape.org/ . Interpretation instructions can be found in Additional file 7. Please note the node pie colours are not available in this file and readers should refer to Fig. 3 and Additional file 5: Figure S2. (CYS 639 kb)

Additional file 7: Description of details relating to line colour, style and width as well as node pie colour to assist with the interpretation of Fig. 3 and Additional file 5: Figure S2 and Additional file 6: Dataset S4. (DOCX 15 kb)

Additional file 8: Figure S3. picto-graph depicting the experimental procedure used for tissue collection, RNA isolation, library construction & sequencing and post sequencing bioinformatics. (PDF 1350 kb)

Additional file 9: Detail methodology for the creation of the sequencing libraries. (DOCX 15 kb)

Additional file 10: Figure S4. Graph of the gene-wise dispersion values; pooled gene-level variance versus mean gene expression levels. Graph indicates a linear relationship. (PNG 12 kb)

## Abbreviations

AT: Apical tip
CoA: Coenzyme A
CSIRO: Commonwealth Scientific Industrial Research Organisation
DNA: Deoxyribonucleic acid
FDR: False discovery rate
FL: Fully expanded leaf
GO: Gene ontology
NADPH: Nicotinamide adenine dinucleotide phosphate
NB-ARC: Nucleotide-binding APAF-1, R proteins and CED-4
PH: Secondary phloem
RNA: Ribonucleic acid
SEA: AgriGo Singular Enrichment Analysis
SS: Severe stress
ST: Primary stem
TAIR10: The Arabidopsis Information Resource
TCA: Tricarboxylic acid cycle
TIR-NBS-LRR: Toll-interleukin-like receptor- nucleotide binding site - C-terminal leucine-rich repeat
UDP: Uridine diphosphate
WW: Well watered
XY: Secondary xylem
