# Supplementary figures and images for: Medium term water deficit elicits distinct transcriptome responses in Eucalyptus species of contrasting environmental origin
Source: BMC Genomics. 2017 Apr 7;18:284. doi: 10.1186/s12864-017-3664-z (PMC5383985; doi:10.1186/s12864-017-3664-z)

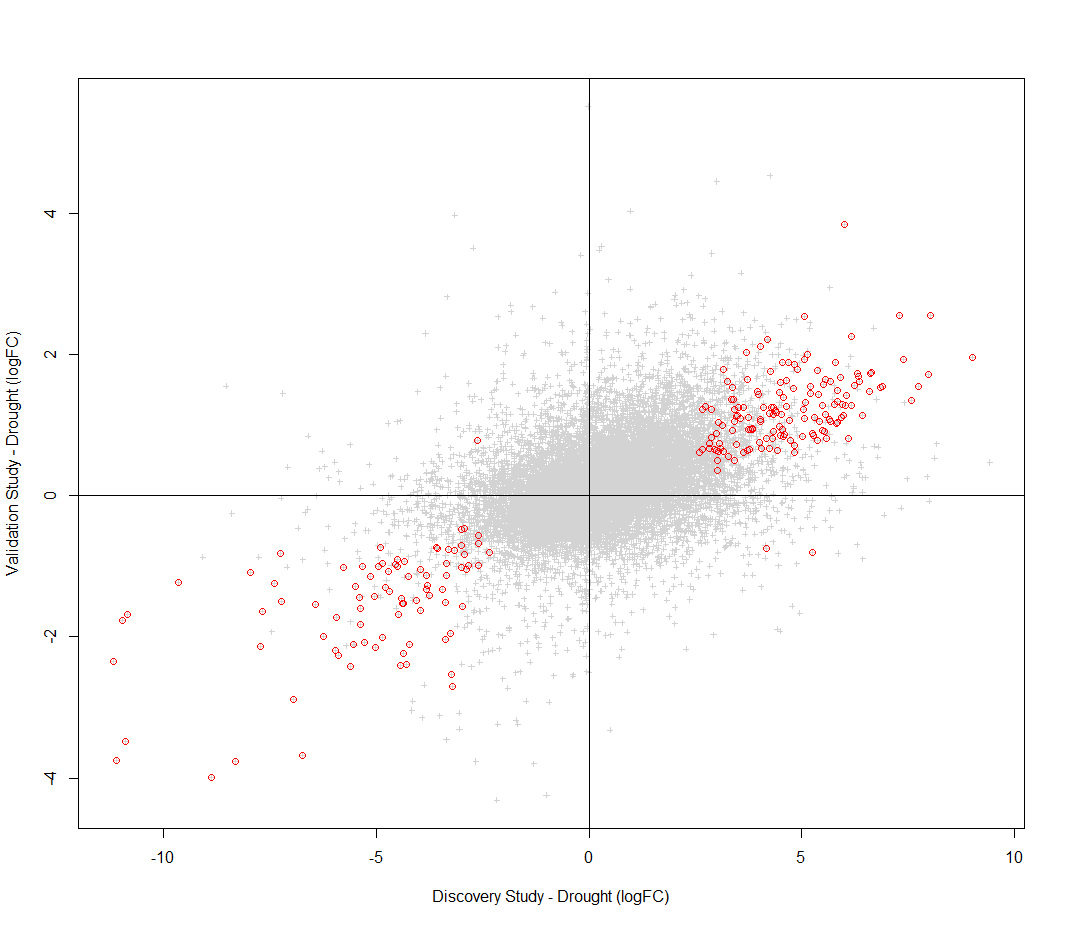

Supplement: Supplementary file 3 — Plot of log fold change in gene expression for leaf and stem tissues between water deficit (SS) treatment or control (WW) treatment as detected in this experiment (X-axis) versus log fold change in gene expression in leaf tissues between water deficit (SS) treatment or control (WW) treatment (Y-axis) as detected in a follow up water deficit experiment on seven eucalypt species (N = 56). Grey (+) symbols represent all genes while red (o) represent genes validated at an FDR of < 0.01 in both experiments. It is clear that validated genes also show responses in the same direction between experiments. (PNG 25 kb) [file 12864_2017_3664_MOESM3_ESM.png]

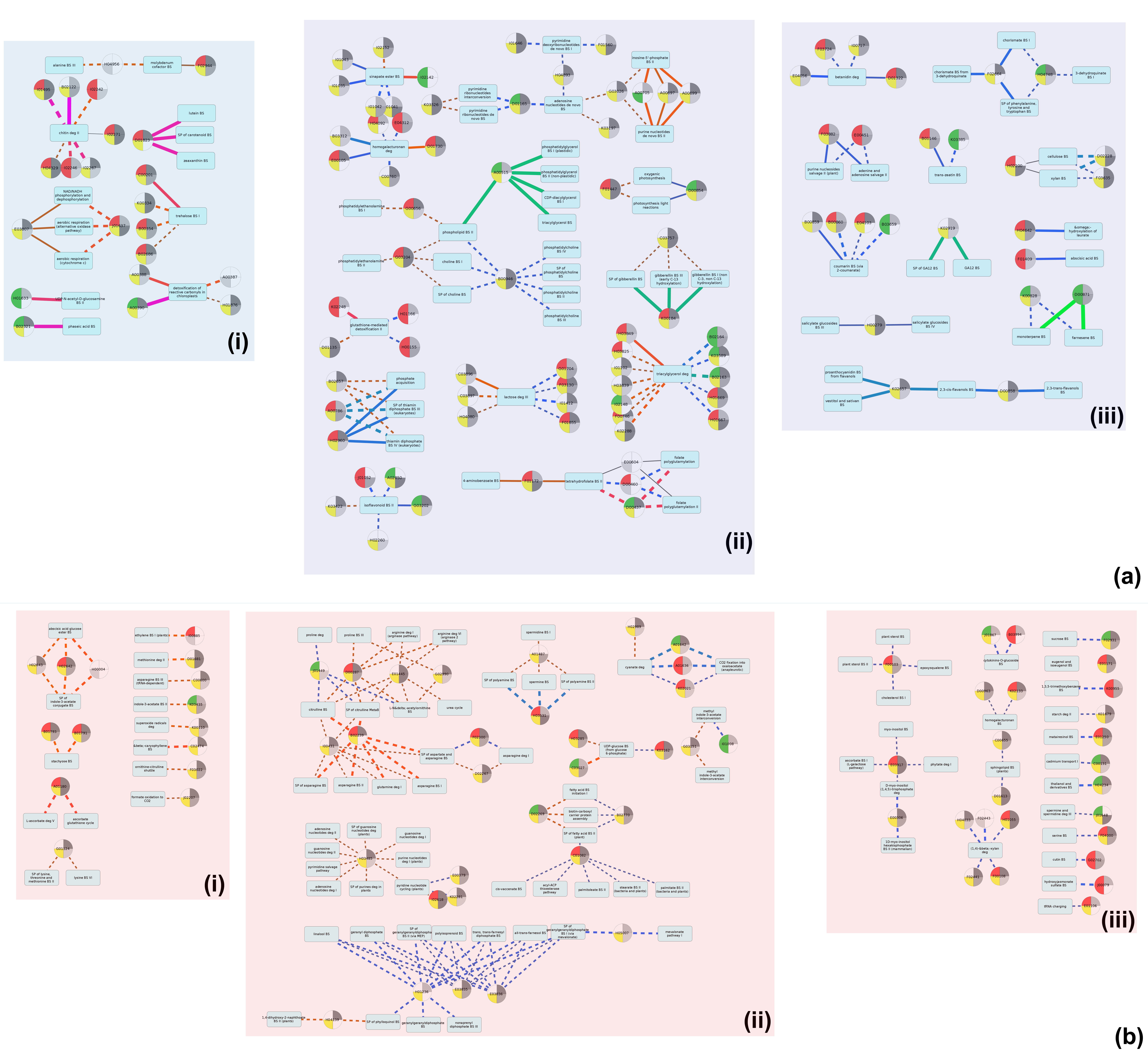

Supplement: Supplementary file 5 — Small unlinked clusters depicting genes with an FDR of <0.2 with known links to pathways in plant metabolic networks. Small unlinked clusters have been arranged according to whether they show a species specific water deficit response (solid lines), with or without a water deficit only treatment response (dashed lines), (a), or water deficit only treatment response (dashed lines) (b). Clusters have been arranged further into those with a higher expression in response to the water deficit treatment (SS) (i), a differential response to treatment where some genes in a cluster show higher expression in response to water deficit treatment (SS) and others to the well watered control (WW) (ii), or higher expression in response to the well watered control only (WW) (iii). (PNG 2743 kb) [file 12864_2017_3664_MOESM5_ESM.png]

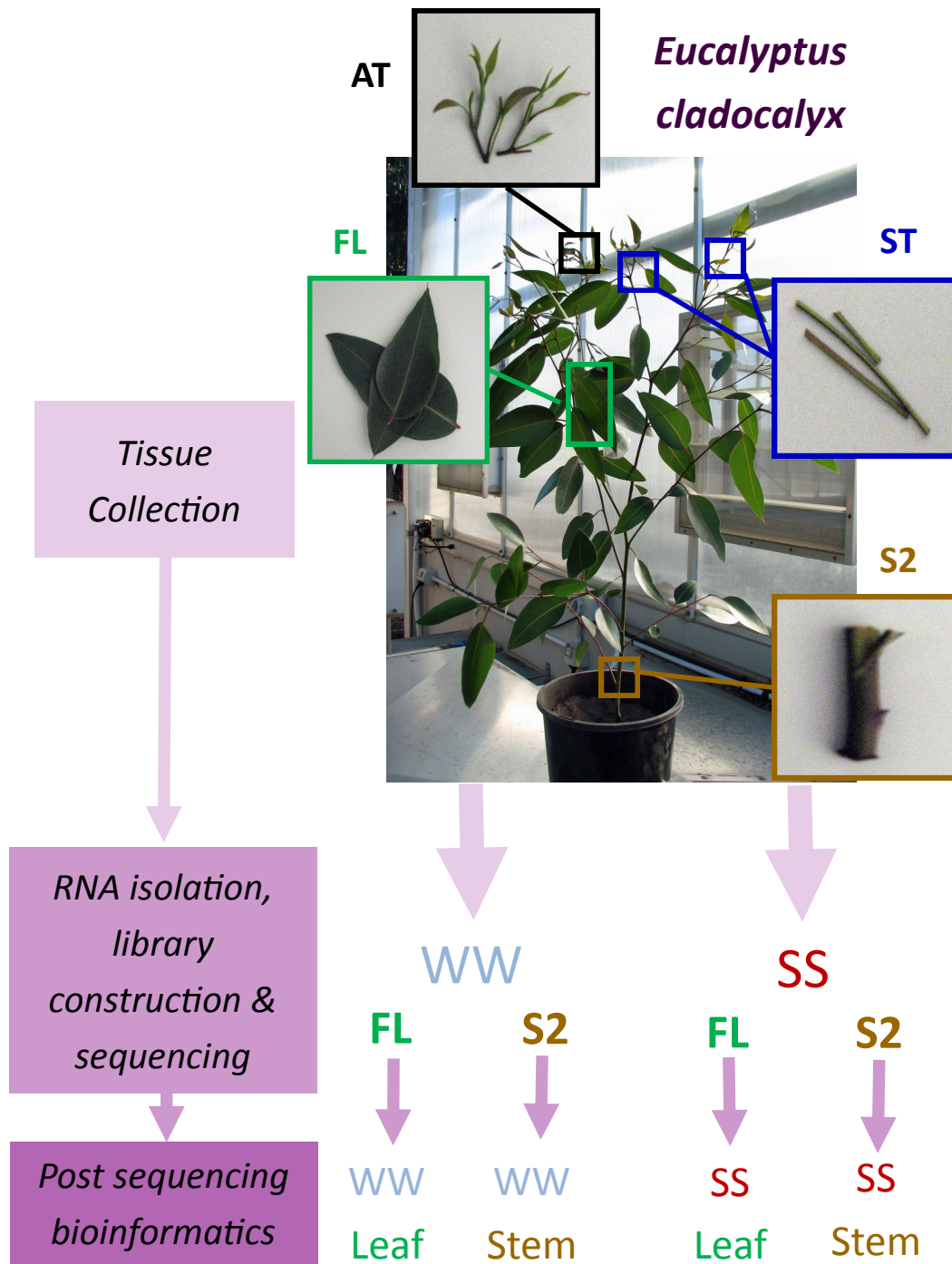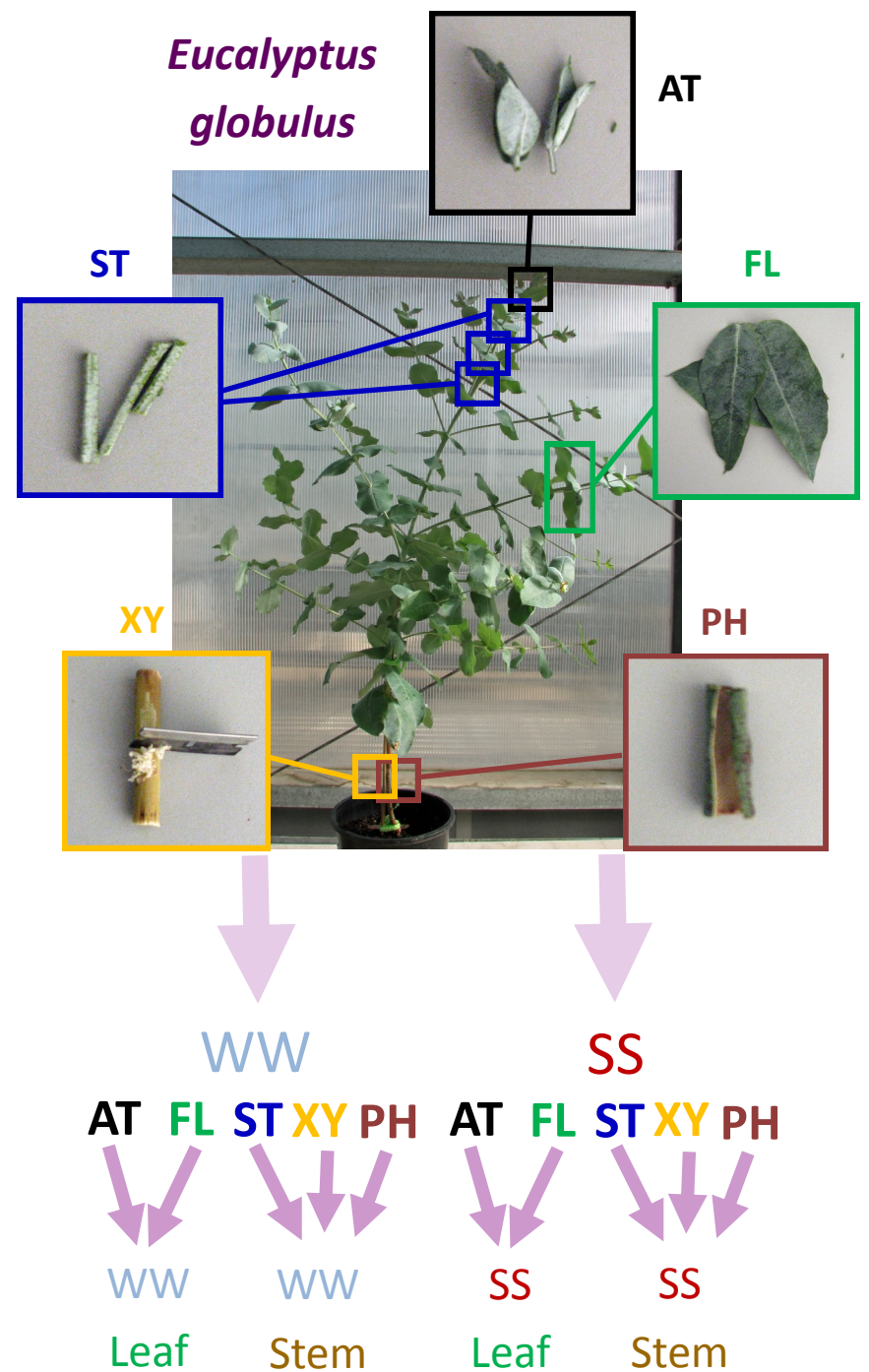

Supplement: Supplementary file 8 — picto-graph depicting the experimental procedure used for tissue collection, RNA isolation, library construction & sequencing and post sequencing bioinformatics. (PDF 1350 kb) [file 12864_2017_3664_MOESM8_ESM.pdf]

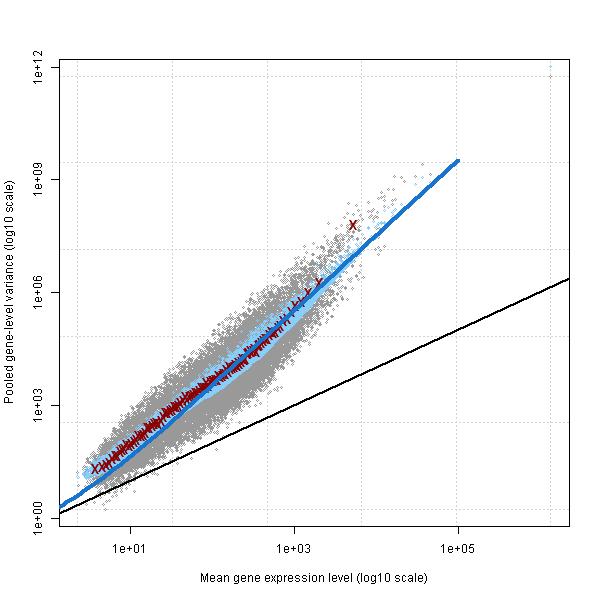

Supplement: Supplementary file 10 — Graph of the gene-wise dispersion values; pooled gene-level variance versus mean gene expression levels. Graph indicates a linear relationship. (PNG 12 kb) [file 12864_2017_3664_MOESM10_ESM.png]
