# Supplementary material for: Medium term water deficit elicits distinct transcriptome responses in Eucalyptus species of contrasting environmental origin
Source: BMC Genomics. 2017 Apr 7;18:284. doi: 10.1186/s12864-017-3664-z (PMC5383985; doi:10.1186/s12864-017-3664-z)
Supplement: Supplementary file 7 — Description of details relating to line colour, style and width as well as node pie colour to assist with the interpretation of Fig. 3 and Additional file 5: Figure S2 and Additional file 6: Dataset S4. (DOCX 15 kb) [file 12864_2017_3664_MOESM7_ESM.docx]

## Cytoscape Visualisation Details

### Explanation of line color, style and width have been created.

**Color** : It is a color gradient defined from the value of the D_Log_FC for each gene. The color order of this gradient is pink, at the lowest Log_FC (SS response) then red, orange, brown, through to grey, blue and finally green at its highest Log_FC(WW response)

**Width** : The edge width is a function of the D_Log_FC column. At the lowest and highest values, the width is maximum. At 0.0, the width is minimum

**Style** : The edge (line) is either solid or dashed. It is solid when the Species_DroughtW_FDR column value is below or equal to 0.2. It is dashed when the Species_DroughtW_FDR column value is above 0.2 and the DroughtW_FDR value is below or equal to 0.2.

### Explanation gene node pie colour.

**Upper-left**: If the maximum value found in a leaf columns (ECFLS, ECFLW, EGATS, EGATW, EGFLS and EGFLW) is at least 2 times higher than the maximum found in a Stem columns (ECS2S, ECS2W, EGSTS, EGSTW, EGXYS, EGXYW, EGPHS and EGPHW), then the colour is green. If it is reverse, the colour is red. If there is no 2 folds difference, the colour is white.

**Upper-right**: This part is a grey gradient about EC coherence. Two stress values are computed. The first one, if ECFLS or ECFLW counts per million (CPM) is above five, then the difference between is further assessed to see if it is above or below zero. Similarly, if ECS2S or ECS2W is above a CPM of five, then the difference between them is assessed to see if it is above or below zero. The colour is determined based on the following parameters; if both tissues respond significantly in the same way it is represented by dark grey, if only one tissue responds significantly it is represented by a light grey and if both are significant but in opposite directions or both are not significant at all it is represented by white.

**Lower-right**: This part is a grey gradient about EG coherence. There are five pairs of EG values ([EGATS and EGATW], [EGFLS and EGFLW], [EGSTS and EGSTW], [EGXYS and EGXYW] and [EGPHS and EGPHW]). The more ‘coherent’ they are, the darker the grey colour is as per the above descriptions.

**Lower-left**: This corresponds to which part of the plant where the gene is more highly expressed. If the gene is expressed equally in both leaf and stem tissues it is represented as yellow. If the gene is more expressed in leaf tissue it is represented as green. If the gene is more highly expressed in stem tissue it is represented as red. Light grey is used when the gene is not significantly expressed in any of these tissues.
