# Supplementary material for: Medium term water deficit elicits distinct transcriptome responses in Eucalyptus species of contrasting environmental origin
Source: BMC Genomics. 2017 Apr 7;18:284. doi: 10.1186/s12864-017-3664-z (PMC5383985; doi:10.1186/s12864-017-3664-z)
Supplement: Supplementary file 9 — Detail methodology for the creation of the sequencing libraries. (DOCX 15 kb) [file 12864_2017_3664_MOESM9_ESM.docx]

Detailed Experimental Procedures for sequencing library preparation

For mSEQ library preparation total RNA was PolyA enriched using the Dynabead mRNA purification kit (Invitrogen, Grand Island, NY, USA) following the manufacturer’s instructions. The enriched RNA was sheared by heating to 94°C in 1x Super Script III Reverse Transcriptase cDNA synthesis buffer (Invitrogen) for 5 minutes and then cooled to 25°C where the remaining buffer components were added (3 µM random hexamers (Invitrogen), 1 mM dNTPs, 10 mM Dithiothreitol and 20 U RNaseOUT (Invitrogen)). Reverse transcription into cDNA was at 42°C for 30 mins and 50°C for 30 mins in a total volume of 20 µL following the addition 20 U Super Script III Reverse Transcriptase (Invitrogen). cDNA synthesis was stopped by heating to 70°C for 15 mins. Second strand synthesis and end polishing followed in a total volume of 150 µL at 16°C for 2.5 hrs with the addition of MBG water, a final concentration of 1x NEB Buffer 2 (New England Biolabs (NEB), Ipswich, MA, USA), 200 µM dNTPs and 40 U DNA Polymerase I (NEB) and 5 U E. coli RNaseH (NEB). The dscDNA was cleaned using a SPRI bead (Beckman-Coulter, Brea, CA, USA) cleanup at a ratio of 1.4:1 following the manufacturer’s instructions. The dscDNA was then dA-Tailed using 5 U Klenow Fragment (NEB E6054AA) in 1x NEB Buffer 2 and 200 µM dNTPs in 25 µL at 37°C for 30 mins and heat inactivated at 65°C for 15 mins. The tailed dscDNA was then ligated with Illumina Y junction adaptor fragments in 30 µL final volume following the addition of 1x T4 DNA ligase reaction buffer (NEB) and 200 U T4 DNA Ligase overnight at 12°C. Ligation reactions were stopped by heating to 65°C for 10 mins and libraries size selected using a 1:1 ratio SPRI cleanup. Selected library fragments were then individually barcoded using primers compatible with Illumina sequencing platforms for tracking PCR (1x Phusion HF buffer, 200 µM dNTPs, 200 µM each primer, 1U Phusion polymerase (Finnzymes, Vantaa, Finland), 1µL 1/1000 SYBR Green I ) in a final volume of 50µL on a STRATAGENE Mx3005 (Agilent Technologies, Santa Clara, CA, USA) using an initial denaturation of 98°C for 30 secs followed by cycling conditions of 98°C for 10 secs, 65°C for 20 secs and 72°C for 1 min until they reached approximately 50% maximum yield (between 10 and 15 cycles). Amplified barcoded libraries were checked for size distribution on a GENECHIP1000 (Agilent Technologies) and titrated by Nanodrop 2000 and qPCR using the KAPA Library Quantification Kit. Libraries where equimolar pooled and sequenced on a GAIIX (Illumina San Diego, CA, USA) using v2 chemistry and a standard 100+8+100 bp sequencing protocol.
